# Supplementary material for: Cathepsin B Protease Facilitates Chikungunya Virus Envelope Protein-Mediated Infection Via Endocytosis or Macropinocytosis
Source: Viruses. 2020 Jul 3;12(7):722. doi: 10.3390/v12070722 (PMC7412492; doi:10.3390/v12070722)
Supplement: Supplementary file 1 [file viruses-12-00722-s001.pdf]

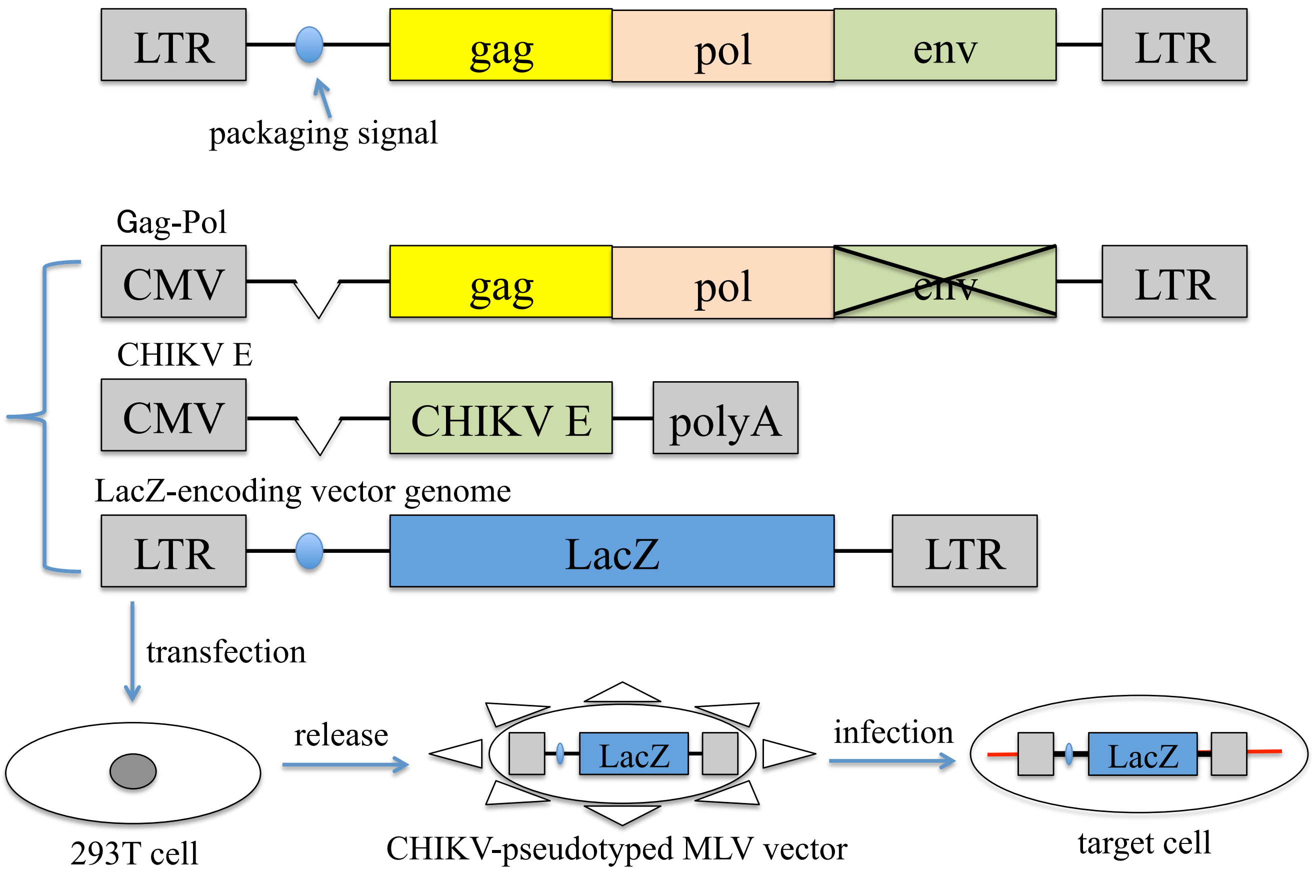

Figure S1 Construction of CHIKV-pseudotyped MLV vector. Structure of the full-length MLV genome is indicated (upper panel). The packaging signal sequence is required for its incorporation into MLV particles. The Gag-Pol expression plasmid for construction of MLV vector lacks the packaging signal and *env* gene. In stead of the deleted MLV *env* gene, CHIKV E expression plasmid is used. The LacZ-encoding MLV vector genome expression plasmid contains the packaging signal sequence. 293T cells were transfected with these three expression plasmids. Only mRNA expressed from the LacZ-encoding MLV vector genome expression plasmid is incorporated into the vector particle. Culture supernatant from the transfected cells contains the CHIKV-pseudotyped MLV vector. Target cells are incubated with the supernatant. The LacZ-encoding MLV vector genome is transmitted and integrated into chromosomal DNA of target cells. LacZ is expressed in infected cells, and X-Gal staining makes the infected cells blue.

A

E3 E2 6K E1

MSLALPVLCL LANTTFPCSQ PPCTPCCYEK EPESTLRMLE DNVMRPGYYQ LLKASLTCSP
HRQRR STKDN FNVYKATRPY LAHCPDCGEG HSCHSPIALE RIRNEATDGT LKIQVSLQIG
IKTDDSHDWT KLRYMDSHTP ADAERAGLLV RTSAPCTITG TMGHFILARC PKGETLTVGF
TDSRKISHTC THPFHHEPPV IGRERFHSRP QHGKELPCST YVQSTAATAE EIEVHMPPDT
PDRTLMTQQS GNVKITVNGQ TVRYKCNCGG SNEGLTTTDK VINNCKIDQC HAAVTNHKNW
QYNSPLVPRN AELGDRKGKI HIPFPLANVT CRVPKARNPT VTYGKNQVTM LLYPDHPTLL
SYRNMGQEPN YHEEWVTHKK EVTLTVPTEG LEVTWGNNEP YKYWPQMSTN GTAHGHPHEI
ILYYYELYPT MTVVIVSVAS FVLLSMVGTA VGMCVCARRR CITPYELTPG ATVPFLLSLL
CCVRTTK AAT YYEAAAYLWN EQQPLFWLQA LIPLAALIVL CNCLKLLPCC CKTLAFLAVM
SIGAHTVSAY EHVTVIPNTV GVPYKTLVNR PGYSPMVLEM ELQSVTLEPT LSLDYITCEY
KTVIPSPYVK CCGTAECKDK SLPDYSCKVF TGVYPFMWGG AYCFCDAENT QLSEAHVEKS
ESCKTEFASA YRAHTASASA KLRVLYQGNN ITVAAYANGD HAVTVKDAKF VVGPMSSAWT
PFDNKIVVYK GDVYNMDYPP FGAGRPGQFG DIQSRTPESK DVYANTQLVL QRPAAGTVHV
PYSQAPSGFK YWLKERGASL QHTAPFGCQI ATNPVRAVNC AVGNIPISID IPDAAFTRVV
DAPSVTDMSC EVPACTHSSD FGGVAIIKYT ASKKGKCAVH SMTNAVTIRE ADVEVEGNSQ
LQISFSTALA SAEFRVQVCS TQVHCAAACH PPKDHIVNYP ASHTTLGVQD ISTTAMSWVQ
KITGGVGLIV AVAALILIVV LCVSFSRH*

B

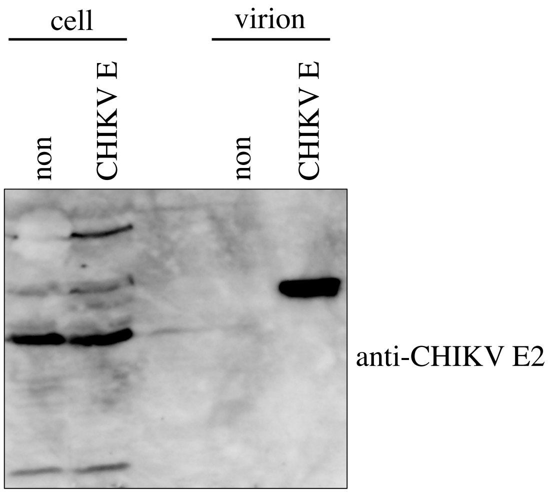

Figure S2 Generation of anti-CHIKV E2 antiserum. (A) The amino acid sequence of CHIKV E protein is indicated. Blue, red, black, and green letters show E3, E2, 6K, and E1 proteins, respectively. A rabbit was inoculated with the underlined peptide. (B) 293T cells were transfected by the MLV Gag-Pol and LacZ-encoding MLV vector genome expression plasmids together with pcDNA3.1 (non) or CHIKV E expression plasmid. Cell lysates and virion pellets prepared from the transfected 293T cells were analyzed by western blotting using the antiserum.
